# Relationship between maternal consumption of fermented foods and the development of the offspring at the age of 3 years: The Japan Environment and Children’s Study

**DOI:** 10.1371/journal.pone.0305535

**Published:** 2024-06-21

**Authors:** Hiroko Hirai, Tomomi Tanaka, Kenta Matsumura, Akiko Tsuchida, Kei Hamazaki, Yuichi Adachi, Hidekuni Inadera, the Japan Environment and Children’s Study Group

**Affiliations:** 1 Faculty of Medicine, Department of Pediatrics, University of Toyama, Toyama, Japan; 2 Toyama Regional Center for JECS, University of Toyama, Toyama, Japan; 3 Faculty of Medicine, Department of Public Health, University of Toyama, Toyama, Japan; 4 Pediatric Allergy Center, Toyama Red Cross Hospital, Toyama, Japan; Universidad San Francisco de Quito, ECUADOR

## Abstract

**Background:**

It is well known that maternal diet affects the development of offspring. Herein, the relationship between maternal intake of fermented foods during pregnancy and offspring development was investigated.

**Methods:**

The diet of 103,060 pregnant women at >4 months of gestation who were enrolled in the Japan Environment and Children’s Study was analyzed. Their intake levels of fermented soybeans (*miso* and *natto*), yogurt, and cheese were investigated. The developmental status of the offspring at 3 years of age was assessed using the Ages and Stages Questionnaires (ASQ-3). Multivariable logistic regression analysis was performed to determine the risk of maternal intake levels of the fermented foods associated with subsequent developmental delay in the offspring.

**Results:**

Intake of cheese was associated with a reduced risk of child developmental delay in all intake level groups from the second quartile onward. Intakes of *miso* and yogurt were associated with a reduced risk of developmental delay in communication skills in the fourth quartile. There was no association between intake of *natto* and developmental delay.

**Conclusion:**

Maternal consumption of fermented foods during pregnancy may reduce the risk of later developmental delay in offspring. It is therefore important to review the mother’s diet for fermented foods during pregnancy. However, further studies are warranted to evaluate the factors influencing the association between diet and offspring development.

## Introduction

Intake of fermented foods affects the regulation of intestinal microbiota and is effective in preventing the progression of various diseases, including diabetes, allergies, depression, obesity, and constipation [1–5]. In addition, previous studies have shown a relationship between autism spectrum disorder or depressive symptoms and gut–brain interaction, as well as between psychiatric symptoms or neurodevelopment and intake of fermented foods [6,7].

Although the intestinal microbiota changes with diet, it is thought that the fetal intestinal microbiota begins to develop in utero and is inherited from the mother [8,9]. This suggests that intake of fermented foods during pregnancy may affect fetal development by improving the intestinal environment. Some fermented foods also contain nutrients that are beneficial to the child’s development and are therefore considered beneficial to child health [10]. Our research group previously investigated the relationship between maternal intake of four fermented foods commonly consumed in Japan—*miso*, *natto*, yogurt, and cheese—during pregnancy and the development of offspring at 1 year of age, and reported a beneficial association [11]. In the present study, we investigated whether the association persisted in the offspring at 3 years of age.

## Methods

The JECS protocol was reviewed and approved by the Ministry of the Environment’s Institutional Review Board on Epidemiological Studies (Ethical Number: No. 100910001) and the ethics committees of all participating institutions.

### Participants

**Fig 1:**
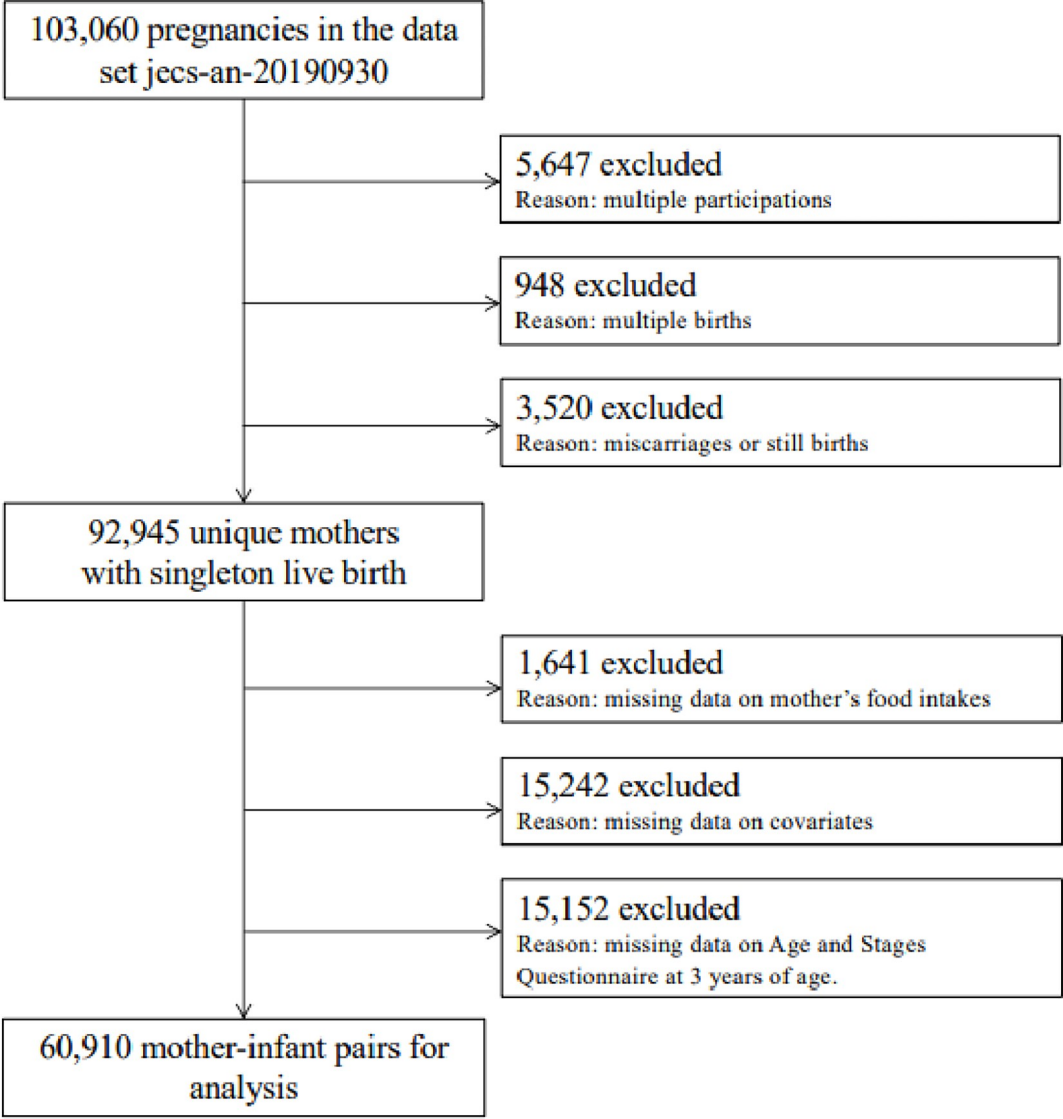
Flow diagram of participant selection. This study included 103,060 pregnant women, 5,647 of whom were excluded due to multiple enrollment, 948 due to multiple pregnancies, 3,520 due to miscarriage or stillbirth, and 32,305 due to lack of information on diet or developmental delay or due to inability to respond to the survey. A total of 60,910 mother–infant pairs were analyzed in the study.

The present study was based on data from the Japan Environment and Children’s Study (JECS), a nationwide birth cohort study that focuses on the relationship of environmental factors with child health and development. The JECS enrolled pregnant women from study areas throughout Japan between January 2011 and March 2014. Details of the JECS design have been reported previously [12–14]. We used data from the jecs-an-20190930 dataset released in October 2019 in the present study. The dataset includes 103,060 pregnancies. We excluded 5,647 due to multiple enrollment, 948 due to multiple pregnancies, 3,520 due to miscarriage or stillbirth, and 32,305 due to lack of information on maternal diet or developmental delay or due to inability to respond to the survey. Thus, a total of 60,910 mother–infant pairs were analyzed in this study (Fig 1). All maternal dietary data were obtained via a self-administered questionnaire on the consumption of fermented foods during pregnancy. Written informed consent for the study was obtained from all participants.

### Fermented foods

The intake of four types of fermented foods (*miso*, *natto*, yogurt, and cheese) by the mothers at >4 months of gestation using the Food Frequency Questionnaire [15]. This semi-quantitative questionnaire contains the following items: name of food, frequency of consumption, and estimated amount consumed in a single meal (weight, nutritional values, and portion size) [16]. These four foods are commonly consumed in Japan.

### Neurodevelopment

The primary outcome was neurodevelopmental status of the offspring at 3 years of age. Neurodevelopment was assessed using the Ages and Stages Questionnaires (ASQ-3). The ASQ-3 is a modified version of the parent-completed child monitoring system, which is a UNICEF-recommended and validated neurodevelopment assessment tool [17,18]. The ASQ consists of 21 age-specific questionnaires administered over time and assesses development in five domains: communication skills, gross motor skills, fine motor skills, problem-solving skills, and social skills. The questionnaire consists of 30 questions: six for each of the five major domains. Each can be answered with “yes,” “no,” “sometimes,” or “not yet.” A score of 10 is given for “yes,” indicating that the child has achieved the item; a score of 5 is given for “sometimes,” indicating that the child is still developing; and a score of 0 is given for “not yet” and “no,” indicating that the child has not yet acquired the ability to achieve the item. If one or two of the six questions were unanswered, a correlation coefficient of 1.2–1.5 was multiplied by the actual number of points earned, and the scores were adjusted to be in the range of 0–60 points; if three or more of the six questions were unanswered, the scores were excluded from the analysis. Participants with scores equal to or below the cut-off values were considered positive cases [19]. The ASQ-3 was completed by mothers for offspring between 35 and 37 months of age.

### Statistical analysis

Data are presented as the mean ± standard deviation or median, unless otherwise stated. To estimate the risk of neurodevelopmental delay with intake of each fermented food, the participants were divided into four groups according to intake level. Odds ratios and 95% confidence intervals were obtained from a multivariate logistic regression analysis. Potential confounders from previous reports included maternal age (<25, 25–<30, 30–<35, ≥35 years), body mass index (<18.5 kg/m^2^, 18.5–25 kg/m^2^, ≥25 kg/m^2^), parity (primipara, multipara), smoking status (never, previously smoked, current smoker), passive smoking (almost never or never, >1 per week), alcohol intake (never, previously drank, current drinker), quartile of physical activity (MET min/week) [20], quartile of folic acid intake (μg), quartile of energy intake (cal), marital status (married [including common-law marriage], single [never married], divorced or widowed), highest education level (≤12, 12–<16, ≥16 years), highest education level of partner (≤12, 12–<16, ≥16 years), employed (yes, no), annual household income (<4 million yen, 4–6 million yen, ˃6 million yen), administration of anti-bacteria medicine (yes, no). Potential mediators were not used as covariates.

## Results

The intake level of each of the four fermented foods consumed during pregnancy was categorized into quartiles: *miso*, 0–24 g, 25–74 g, 75–145 g, and 147–2,063 g; *natto*, 0–1.7 g, 3.3–5.4 g, 10.7–12.5 g, and 16.1−600.0 g; yogurt, 0–8 g, 12–26 g, 30–90 g, and 94–1,440 g; and cheese, 0–0.7 g, 1.3–2.0 g, 2.1–4.3 g, and 5.0–240.0 g. The characteristics of each quartile for cheese intake during pregnancy are shown in Table 1. The characteristics of the other fermented foods are shown in S1–S3 Tables in S1 File. Compared with mothers who consumed less yogurt, those who consumed more yogurt had a higher level of education and annual income, and a higher percentage were nulliparas. Further, their partners had a higher level of education and a lower percentage were smokers or passive smokers. For all four fermented foods, higher intake groups also had higher energy and folic acid intakes compared with the lowest intake group.

**Table 1:** Demographic and obstetric characteristics of participants (Cheese:n = 60,910).

Variable		Cheese Intake (unit needed to be checked)							
		Q1 (0.0–0.7 g)		Q2 (1.3–2.0 g)		Q3 (2.1–4.3 g)		Q4 (5.0–240.0 g)	
		N (%)		N (%)		N (%)		N (%)	
Subtotal		14,744	(24.2)	13,981	(23.0)	17,463	(28.7)	14,722	(24.2)
	Age, y								
	<25	1,628	(11.0)	1,279	(9.2)	1,152	(6.6)	760	(5.2)
	25–<30	4,496	(30.5)	4,202	(30.1)	4,698	(26.9)	3,598	(24.4)
	30–<35	5,118	(34.7)	4,990	(35.7)	6,549	(37.5)	5,632	(38.3)
	≥35	3,502	(23.8)	3,510	(25.1)	5,064	(29.0)	4,732	(32.1)
	Body mass index, kg/m^2^								
	<18.5	2,403	(16.3)	2,220	(15.9)	2,801	(16.0)	2,344	(15.9)
	18.5 –<25	10,624	(72.1)	10,324	(73.8)	13,175	(75.5)	11,123	(75.6)
	≥25	1,717	(11.7)	1,437	(10.3)	1,487	(8.5)	1,255	(8.5)
	Parity								
	Primipara	7,038	(47.7)	6,543	(46.8)	7,322	(41.9)	5,985	(40.7)
	Multipara	7,706	(52.3)	7,438	(53.2)	10,141	(58.1)	8,737	(59.4)
	Smoking status								
	Never	8,275	(56.1)	8,409	(60.2)	10,994	(63.0)	9,324	(63.3)
	Former	5,770	(39.1)	5,055	(36.2)	6,010	(34.4)	5,026	(34.1)
	Current	699	(4.7)	517	(3.7)	459	(2.6)	372	(2.5)
	Passive smoking								
	No	8,893	(60.3)	8,868	(63.4)	11,741	(67.2)	10,048	(68.3)
	Yes	5,851	(39.7)	5,113	(36.6)	5,722	(32.8)	4,674	(31.8)
	Alcohol intake								
	Never	5,361	(36.4)	4,641	(33.2)	5,703	(32.7)	4,799	(32.6)
	Former	9,071	(61.5)	8,968	(64.1)	11,278	(64.6)	9,463	(64.3)
	Current	312	(2.1)	372	(2.7)	482	(2.8)	460	(3.1)
	Physical activity								
	Q1	3,930	(26.7)	3,452	(24.7)	4,063	(23.3)	3,273	(22.2)
	Q2	3,533	(24.0)	3,485	(24.9)	4,528	(25.9)	3,778	(25.7)
	Q3	3,524	(23.9)	3,661	(26.2)	4,773	(27.3)	4,024	(27.3)
	Q4	3,757	(25.5)	3,383	(24.2)	4,099	(23.5)	3,647	(24.8)
	Quintile of folic acid intake, μg								
	Q1	5,764	(39.1)	4,143	(29.6)	3,123	(17.9)	1,390	(9.4)
	Q2	3,619	(24.6)	4,044	(28.9)	4,777	(27.4)	2,919	(19.8)
	Q3	2,977	(20.2)	3,260	(23.3)	5,083	(29.1)	4,391	(29.8)
	Q4	2,384	(16.2)	2,534	(18.1)	4,480	(25.7)	6,022	(40.9)
	Energy intake								
	Q1	5,708	(38.7)	4,035	(28.9)	3,213	(18.4)	1,537	(10.4)
	Q2	3,883	(26.3)	4,134	(29.6)	4,823	(27.6)	2,941	(20.0)
	Q3	2,823	(19.2)	3,354	(24.0)	5,158	(29.5)	4,216	(28.6)
	Q4	2,330	(15.8)	2,458	(17.6)	4,269	(24.5)	6,028	(41.0)
	Marital status								
	Married	14,091	(95.6)	13,463	(96.3)	16,974	(97.2)	14,372	(97.6)
	Single	526	(3.6)	431	(3.1)	397	(2.3)	290	(2.0)
	Divorced or widowed	127	(0.9)	87	(0.6)	92	(0.5)	60	(0.4)
	Highest education level, y								
	≤12	6,001	(40.7)	4,853	(34.7)	5,086	(29.1)	3,802	(25.8)
	12 –<16	6,086	(41.3)	5,951	(42.6)	7,810	(44.7)	6,547	(44.5)
	≥16	2,657	(18.0)	3,177	(22.7)	4,567	(26.2)	4,373	(29.7)
	Highest education level of							
	partner, y								
	≤12	7,031	(47.7)	5,999	(42.9)	6,690	(38.3)	5,284	(35.9)
	12 –<16	3,307	(22.4)	3,223	(23.1)	4,076	(23.3)	3,338	(22.7)
	≥16	4,406	(29.9)	4,759	(34.0)	6,697	(38.4)	6,100	(41.4)
	Employed								
	No	6,362	(43.2)	6,204	(44.4)	8,126	(46.5)	6,979	(47.4)
	Yes	8,382	(56.9)	7,777	(55.6)	9,337	(53.5)	7,743	(52.6)
	Annual household income,								
	million yen								
	<4	6,547	(44.4)	5,401	(38.6)	6,302	(36.1)	4,942	(33.6)
	4 –<6	4,628	(31.4)	4,757	(34.0)	6,042	(34.6)	5,163	(35.1)
	≥6	3,569	(24.2)	3,823	(27.3)	5,119	(29.3)	4,617	(31.4)
	Anti-bacteria medicine								
	No	13,189	(89.5)	12,473	(89.2)	15,574	(89.2)	13,127	(89.2)
	Yes	1,555	(10.6)	1,508	(10.8)	1,889	(10.8)	1,595	(10.8)

Table 2 shows the ORs of neurodevelopmental delay in each domain, as assessed by the ASQ-3, according to the quartiles of fermented food intake relative to the first quartile. Multivariable logistic regression analysis revealed that cheese intake during pregnancy significantly reduced the risk of neurodevelopmental delay in offspring at 3 years of age in all five domains.

**Table 2:** Adjusted odds ratios (AORs) and 95% confidence intervals (95% CIs) for developmental delay in each area assessed using ASQ-3 according to fermented food intakes during the pregnancy.

		Quartile				
		Q1	Q2	Q3	Q4	*p* for
			AOR [95% CI]	AOR [95% CI]	AOR [95% CI]	trend
Cheese						
	Communication	1.00 (Ref.)	0.84 [0.76–0.93]	0.82 [0.74–0.90]	0.82 [0.74–0.91]	< .001
	Gross motor	1.00 (Ref.)	0.79 [0.71–0.89]	0.83 [0.74–0.92]	0.84 [0.75–0.94]	.006
	Fine motor	1.00 (Ref.)	0.87 [0.78–0.96]	0.81 [0.73–0.89]	0.78 [0.70–0.87]	< .001
	Problem solving	1.00 (Ref.)	0.82 [0.73–0.92]	0.80 [0.71–0.89]	0.77 [0.68–0.87]	< .001
	Personal-social	1.00 (Ref.)	0.76 [0.67–0.87]	0.79 [0.70–0.90]	0.81 [0.70–0.92]	.002
Yogurt							
	Communication	1.00 (Ref.)	0.91 [0.82–1.00]	0.93 [0.84–1.03]	0.89 [0.80–0.98]	.034
	Gross motor	1.00 (Ref.)	1.02 [0.91–1.15]	1.03 [0.92–1.16]	0.95 [0.85–1.06]	.358
	Fine motor	1.00 (Ref.)	1.04 [0.93–1.16]	1.04 [0.94–1.16]	1.01 [0.90–1.12]	.935
	Problem solving	1.00 (Ref.)	0.96 [0.85–1.08]	0.94 [0.83–1.06]	1.01 [0.90–1.14]	.871
	Personal-social	1.00 (Ref.)	1.02 [0.90–1.16]	0.93 [0.82–1.06]	0.95 [0.84–1.08]	.277
Miso soup						
	Communication	1.00 (Ref.)	1.01 [0.92–1.11]	0.96 [0.87–1.05]	0.87 [0.79–0.96]	.004
	Gross motor	1.00 (Ref.)	0.96 [0.86–1.08]	0.94 [0.85–1.04]	0.96 [0.86–1.07]	.343
	Fine motor	1.00 (Ref.)	1.02 [0.92–1.13]	0.97 [0.88–1.07]	0.98 [0.88–1.09]	.530
	Problem solving	1.00 (Ref.)	1.01 [0.90–1.13]	1.03 [0.92–1.14]	0.97 [0.86–1.08]	.670
	Personal-social	1.00 (Ref.)	0.98 [0.86–1.11]	0.91 [0.81–1.03]	0.94 [0.83–1.06]	.182
Natto						
	Communication	1.00 (Ref.)	0.92 [0.83–1.02]	0.92 [0.83–1.02]	1.01 [0.91–1.13]	.790
	Gross motor	1.00 (Ref.)	0.95 [0.85–1.07]	0.87 [0.77–0.97]	1.01 [0.89–1.14]	.780
	Fine motor	1.00 (Ref.)	0.87 [0.78–0.97]	0.85 [0.76–0.95]	0.89 [0.80–1.00]	.054
	Problem solving	1.00 (Ref.)	0.92 [0.81–1.04]	0.93 [0.82–1.04]	0.95 [0.83–1.08]	.468
	Personal-social	1.00 (Ref.)	0.93 [0.81–1.06]	0.85 [0.75–0.97]	0.95 [0.82–1.09]	.272

## Discussion

When mothers consumed ≥1.3 g of cheese daily during pregnancy, the risk of motor and neurodevelopmental delays in their offspring at the age of 3 years was significantly reduced. Fermented foods are expected to have health-promoting effects due to fermentation by various microorganisms, which enhance the nutritional value of the foods compared with their original non-fermented state. The health-promoting effects of fermented foods may be caused by changes that occur when they are consumed as a meal [21]. There are many reports on the relationship between consumption of fermented foods and neurodevelopment, and gut–brain interactions have attracted increasing research interest recently [6]. In addition to fermented foods, Hamazaki et al. [22], Julvez et al. [23], and Bolduc et al. [24] reported positive correlations between maternal intake of fish and fruits during pregnancy and offspring development. There are also numerous reports on the relationship between intake of vitamins (including folic acid) and trace elements, such as iron, and offspring development [25–29]. Our research group previously found a beneficial association between maternal intake during pregnancy of the same four fermented foods that we assessed in the present study–(*miso*, *natto*, yogurt, and cheese) and the development of the offspring at 1 year of age [11]. The present study is the first to evaluate this association in offspring at the age of 3 years. The mechanisms of this association might be explained by gut–brain interactions mediated by the intestinal microbiota, as described above. Development of the intestinal microbiota begins in utero with the microorganisms of the maternal microbiota [8,9]. Although the fetal gut is sterile, the prototype of subsequent intestinal microbiota is formed within the first week after birth, and the predominant bacterial groups change with growth [30]. Accordingly, maternal intestinal microbiota at the time of birth has a great influence on the composition of the postnatal intestinal microbiota of infants. Thus, improving the intestinal environment of pregnant women through intake of fermented foods could be beneficial to the health of the fetus.

The intestinal microflora affect the nervous system through the action of neurotransmitters produced by various microorganisms and also has anti-inflammatory effects [31–33]. *Bifidobacterium* spp., which are found in high proportions in the gut throughout life, produce the inhibitory neurotransmitter GABA and play an important role in early infant development [6,34]. Because GABA suppresses nerve excitation, the presence of *Bifidobacterium* spp. in the gut may affect the mental activity and behavior of children. Disruption of the intestinal environment, so-called dysbiosis, also promotes intestinal inflammation, which is thought to increase intestinal permeability and facilitate the transfer of toxic neurotransmitters to the central nervous system. It has been suggested that the underlying mechanism of autism spectrum disorders might be brain inflammation associated with increased inflammatory cytokines such as IL-6 and TNF-α [31–33]. Although there are presently no established therapies, some animal studies have reported that administration of probiotics improved symptoms in autistic spectrum disorders, and interventions to improve the gut environment may also be effective in improving neurological symptoms [35,36].

Many studies have reported the effects of the gut microbiota on the behavior and mental states of the host, but it is difficult to consider the gut microbiota as the underlying cause of developmental disorders. It is speculated that improving the gut microbiota may be effective in alleviating the symptoms of developmental disorders in predisposed children.

In this study, there were differences in the results of the four fermented foods investigated. Only cheese was associated with a reduced risk of developmental delay in all five domains studied. Previous studies have suggested that intake of fermented dairy products may be effective in preventing cognitive decline in Alzheimer’s disease, and some fermented dairy products are thought to influence cognitive function [37]. These findings may be explained by differences in the function of the probiotics and nutrients in each fermented food. Various microorganisms are involved in the production of fermented foods: *Aspergillus* mold in *miso*, *Bacillus* in *natto*, and *Lactobacillus* spp. in yogurt and cheese. Although the effects of individual microorganisms on development are unclear, each fermented food has a variety of different health-promoting effects. *Miso* is reported to be effective in preventing hypertension and elevating blood sugar [38]. *Natto* is effective in inhibiting the growth of *Clostridium* spp. and regulating intestinal microbiota [39–41]. Some *Lactobacillus* spp. are known to improve the overall intestinal microbiota composition. Some *Bifidobacterium* spp. increase the counts of *Bifidobacterium* and *Lactobacillus* spp. [34]. In addition, the nutritional components differ in each fermented food [10]. Nutrients such as iron, zinc, long-chain fatty acids, and tryptophan are associated with development [42,43] and are essential in normal neurodevelopment and the differentiation of some brain cells. These nutrients may be transferred from mother to child and affect postnatal development. Regarding the nutritional composition of the four fermented foods investigated in this study, cheese has lower amounts of iron and long-chain fatty acids but higher amounts of protein, zinc, and tryptophan, according to the Japanese standard tables of food composition [44]. However, even small amounts of cheese (≥1.3 g daily) had observable effects. Because a piece of processed cheese sold in Japan weighs about 15–20 g, the effect was observable with a relatively small intake. It is unclear whether the effects of cheese intake on development may be direct, as seen with individual nutrients.

In the group with high cheese intake, the education level of the mother and her partner was higher, and the rate of passive smoking was lower than in the group with the lowest cheese intake, suggesting that there may be factors such as high health consciousness that could not be adjusted for. In the future, it may be possible to clarify the relationship between diet and development by directly examining the intestinal microbiota of mother–infant pairs.

This study has some limitations. First, the manufacturer of each food was not investigated and detailed ingredients were not available. Second, the microorganisms and nutritional components in the same food were not standardized. Third, the intake levels during pregnancy were not strictly measured because the survey was conducted using a questionnaire. Fourth, the data analyzed was obtained only from mothers who had been recruited and consented to participate in the JECS while pregnant at the participating maternity units. Therefore, there may be a bias in which only women who were amenable to participating in the JECS were analyzed in the present study. Finally, in addition to the influence of the maternal diet during pregnancy, the offspring’s diet after birth may also influence neurodevelopment. Furthermore, although the results of this study suggest that the intestinal microbiota is involved, the actual composition of the intestinal microbiota of the mothers and their children was not investigated. Further studies are warranted to address these limitations.

## Conclusion

This study on the relationship between maternal intake of fermented foods during pregnancy and offspring development found the risk of neurodevelopmental delay at the age of 3 years was reduced in the offspring of mothers who consumed more cheese. However, it is possible that the child’s development might have been influenced by the child’s own diet and lifestyle, and thus further studies are needed.

## Supporting information

S1 File S1-S3 Tables shows the demographic and obstetric characteristics of participants for miso, yogurt, and natto, respectively. (DOCX)
