# Supplementary material for: Relationship between maternal consumption of fermented foods and the development of the offspring at the age of 3 years: The Japan Environment and Children’s Study
Source: PLoS One. 2024 Jun 21;19(6):e0305535. doi: 10.1371/journal.pone.0305535 (PMC11192395; doi:10.1371/journal.pone.0305535)
Supplement: S1 File — (DOCX) [file pone.0305535.s001.docx]

|  | Supplemental Table1.Demographic and obstetric characteristics of participants (Miso:n=60,910) | | | | | | | | | | | | | | | |
| --- | --- | --- | --- | --- | --- | --- | --- | --- | --- | --- | --- | --- | --- | --- | --- | --- |
| Variable | | Miso Soup Intake (unit needed to be checked) | | | | | | | | | | |  |  |  |  |
|  |  | Q1 (0–24 g) | | |  | Q2 (25–74 g) | | |  | Q3 (75–145 g) | | |  | Q4 (147–2,063 g) | | |
|  |  | N (%) | | |  | N (%) | | |  | N (%) | | |  | N (%) | | |
| Subtotal | | 15,660 |  | (25.7) |  | 12,935 |  | (21.2) |  | 17,092 |  | (28.1) |  | 15,223 |  | (25.0) |
|  | Age, y |  |  |  |  |  |  |  |  |  |  |  |  |  |  |  |
|  | <25 | 1,406 |  | (9.0) |  | 1,151 |  | (8.9) |  | 1,116 |  | (6.5) |  | 1,146 |  | (7.5) |
|  | 25–<30 | 4,483 |  | (28.6) |  | 3,721 |  | (28.8) |  | 4,653 |  | (27.2) |  | 4,137 |  | (27.2) |
|  | 30–<35 | 5,584 |  | (35.7) |  | 4,673 |  | (36.1) |  | 6,401 |  | (37.5) |  | 5,631 |  | (37.0) |
|  | ≥35 | 4,187 |  | (26.7) |  | 3,390 |  | (26.2) |  | 4,922 |  | (28.8) |  | 4,309 |  | (28.3) |
|  | Body mass index, kg/m2 |  |  |  |  |  |  |  |  |  |  |  |  |  |  |  |
|  | <18.5 | 2,454 |  | (15.7) |  | 2,006 |  | (15.5) |  | 2,830 |  | (16.6) |  | 2,478 |  | (16.3) |
|  | 18.5 – <25 | 11,492 |  | (73.4) |  | 9,681 |  | (74.8) |  | 12,800 |  | (74.9) |  | 11,273 |  | (74.1) |
|  | ≥25 | 1,714 |  | (11.0) |  | 1,248 |  | (9.7) |  | 1,462 |  | (8.6) |  | 1,472 |  | (9.7) |
|  | Parity |  |  |  |  |  |  |  |  |  |  |  |  |  |  |  |
|  | Primipara | 7,721 |  | (49.3) |  | 5,866 |  | (45.4) |  | 7,251 |  | (42.4) |  | 6,050 |  | (39.7) |
|  | Multipara | 7,939 |  | (50.7) |  | 7,069 |  | (54.7) |  | 9,841 |  | (57.6) |  | 9,173 |  | (60.3) |
|  | Smoking status |  |  |  |  |  |  |  |  |  |  |  |  |  |  |  |
|  | Never | 9,278 |  | (59.3) |  | 7,929 |  | (61.3) |  | 10,522 |  | (61.6) |  | 9,273 |  | (60.9) |
|  | Former | 5,742 |  | (36.7) |  | 4,564 |  | (35.3) |  | 6,090 |  | (35.6) |  | 5,465 |  | (35.9) |
|  | Current | 640 |  | (4.1) |  | 442 |  | (3.4) |  | 480 |  | (2.8) |  | 485 |  | (3.2) |
|  | Passive smoking |  |  |  |  |  |  |  |  |  |  |  |  |  |  |  |
|  | No | 10,001 |  | (63.9) |  | 8,280 |  | (64.0) |  | 11,361 |  | (66.5) |  | 9,908 |  | (65.1) |
|  | Yes | 5,659 |  | (36.1) |  | 4,655 |  | (36.0) |  | 5,731 |  | (33.5) |  | 5,315 |  | (34.9) |
|  | Alcohol intake |  |  |  |  |  |  |  |  |  |  |  |  |  |  |  |
|  | Never | 5,496 |  | (35.1) |  | 4,336 |  | (33.5) |  | 5,674 |  | (33.2) |  | 4,998 |  | (32.8) |
|  | Former | 9,769 |  | (62.4) |  | 8,231 |  | (63.6) |  | 10,959 |  | (64.1) |  | 9,821 |  | (64.5) |
|  | Current | 395 |  | (2.5) |  | 368 |  | (2.8) |  | 459 |  | (2.7) |  | 404 |  | (2.7) |
|  | Physical activity |  |  |  |  |  |  |  |  |  |  |  |  |  |  |  |
|  | Q1 | 3,890 |  | (24.8) |  | 2,947 |  | (22.8) |  | 3,956 |  | (23.2) |  | 3,925 |  | (25.8) |
|  | Q2 | 3,795 |  | (24.2) |  | 3,298 |  | (25.5) |  | 4,414 |  | (25.8) |  | 3,817 |  | (25.1) |
|  | Q3 | 4,104 |  | (26.2) |  | 3,498 |  | (27.0) |  | 4,741 |  | (27.7) |  | 3,639 |  | (23.9) |
|  | Q4 | 3,871 |  | (24.7) |  | 3,192 |  | (24.7) |  | 3,981 |  | (23.3) |  | 3,842 |  | (25.2) |
|  | Quintile of folic acid intake, μg | | | |  |  |  |  |  |  |  |  |  |  |  |  |
|  | Q1 | 5,293 |  | (33.8) |  | 3,390 |  | (26.2) |  | 3,440 |  | (20.1) |  | 2,297 |  | (15.1) |
|  | Q2 | 4,073 |  | (26.0) |  | 3,484 |  | (26.9) |  | 4,445 |  | (26.0) |  | 3,357 |  | (22.1) |
|  | Q3 | 3,493 |  | (22.3) |  | 3,270 |  | (25.3) |  | 4,743 |  | (27.8) |  | 4,205 |  | (27.6) |
|  | Q4 | 2,801 |  | (17.9) |  | 2,791 |  | (21.6) |  | 4,464 |  | (26.1) |  | 5,364 |  | (35.2) |
|  | Energy intake |  |  |  |  |  |  |  |  |  |  |  |  |  |  |  |
|  | Q1 | 5,114 |  | (32.7) |  | 3,359 |  | (26.0) |  | 3,626 |  | (21.2) |  | 2,394 |  | (15.7) |
|  | Q2 | 4,103 |  | (26.2) |  | 3,442 |  | (26.6) |  | 4,525 |  | (26.5) |  | 3,711 |  | (24.4) |
|  | Q3 | 3,320 |  | (21.2) |  | 3,276 |  | (25.3) |  | 4,706 |  | (27.5) |  | 4,249 |  | (27.9) |
|  | Q4 | 3,123 |  | (19.9) |  | 2,858 |  | (22.1) |  | 4,235 |  | (24.8) |  | 4,869 |  | (32.0) |
|  | Marital status |  |  |  |  |  |  |  |  |  |  |  |  |  |  |  |
|  | Married | 15,043 |  | (96.1) |  | 12,464 |  | (96.4) |  | 16,596 |  | (97.1) |  | 14,797 |  | (97.2) |
|  | Single | 500 |  | (3.2) |  | 394 |  | (3.1) |  | 410 |  | (2.4) |  | 340 |  | (2.2) |
|  | Divorced or widowed | 117 |  | (0.8) |  | 77 |  | (0.6) |  | 86 |  | (0.5) |  | 86 |  | (0.6) |
|  | Highest education  level,y | | |  |  |  |  |  |  |  |  |  |  |  |  |  |
|  | ≤12 | 5,280 |  | (33.7) |  | 4,109 |  | (31.8) |  | 5,218 |  | (30.5) |  | 5,135 |  | (33.7) |
|  | 12 – <16 | 6,707 |  | (42.8) |  | 5,677 |  | (43.9) |  | 7,444 |  | (43.6) |  | 6,566 |  | (43.1) |
|  | ≥16 | 3,673 |  | (23.5) |  | 3,149 |  | (24.3) |  | 4,430 |  | (25.9) |  | 3,522 |  | (23.1) |
|  | Highest education level of |  |  |  |  |  |  |  |  |  |  |  |  |  |  |  |
|  | partner, y |  |  |  |  |  |  |  |  |  |  |  |  |  |  |  |
|  | ≤12 | 6,607 |  | (42.2) |  | 5,251 |  | (40.6) |  | 6,614 |  | (38.7) |  | 6,532 |  | (42.9) |
|  | 12 – <16 | 3,616 |  | (23.1) |  | 2,961 |  | (22.9) |  | 3,967 |  | (23.2) |  | 3,400 |  | (22.3) |
|  | ≥16 | 5,437 |  | (34.7) |  | 4,723 |  | (36.5) |  | 6,511 |  | (38.1) |  | 5,291 |  | (34.8) |
|  | Employed |  |  |  |  |  |  |  |  |  |  |  |  |  |  |  |
|  | No | 6,864 |  | (43.8) |  | 5,926 |  | (45.8) |  | 8,008 |  | (46.9) |  | 6,873 |  | (45.2) |
|  | Yes | 8,796 |  | (56.2) |  | 7,009 |  | (54.2) |  | 9,084 |  | (53.2) |  | 8,350 |  | (54.9) |
|  | Annual household income, |  |  |  |  |  |  |  |  |  |  |  |  |  |  |  |
|  | million yen |  |  |  |  |  |  |  |  |  |  |  |  |  |  |  |
|  | <4 | 6,276 |  | (40.1) |  | 5,018 |  | (38.8) |  | 6,205 |  | (36.3) |  | 5,693 |  | (37.4) |
|  | 4 – <6 | 5,108 |  | (32.6) |  | 4,360 |  | (33.7) |  | 5,859 |  | (34.3) |  | 5,263 |  | (34.6) |
|  | ≥6 | 4,276 |  | (27.3) |  | 3,557 |  | (27.5) |  | 5,028 |  | (29.4) |  | 4,267 |  | (28.0) |
|  | Anti-bacteria medicine | |  |  |  |  |  |  |  |  |  |  |  |  |  |  |
|  | No | 14,077 |  | (89.9) |  | 11,603 |  | (89.7) |  | 15,235 |  | (89.1) |  | 13,448 |  | (88.3) |
|  | Yes | 1,583 |  | (10.1) |  | 1,332 |  | (10.3) |  | 1,857 |  | (10.9) |  | 1,775 |  | (11.7) |

|  | Supplemental Table2 .Demographic and obstetric characteristics of participants(Yogurt:n=60,910) | | | | | | | | | | | | | | | |
| --- | --- | --- | --- | --- | --- | --- | --- | --- | --- | --- | --- | --- | --- | --- | --- | --- |
| Variable | | Yogurt Intake (unit needed to be checked) | | | | | | | | |  |  |  |  |  |  |
|  |  | Q1 (0–8 g) | | |  | Q2 (12–26 g) | | |  | Q3 (30–90 g) | | |  | Q4 (94–1,440 g) | | |
|  |  | N (%) | | |  | N (%) | | |  | N (%) | | |  | N (%) | | |
| Subtotal | | 16,057 |  | (26.4) |  | 12,874 |  | (21.1) |  | 14,218 |  | (23.3) |  | 17,761 |  | (29.2) |
|  | Age, y |  |  |  |  |  |  |  |  |  |  |  |  |  |  |  |
|  | <25 | 1,865 |  | (11.6) |  | 1,091 |  | (8.5) |  | 980 |  | (6.9) |  | 883 |  | (5.0) |
|  | 25–<30 | 4,837 |  | (30.1) |  | 3,618 |  | (28.1) |  | 3,912 |  | (27.5) |  | 4,627 |  | (26.1) |
|  | 30–<35 | 5,509 |  | (34.3) |  | 4,708 |  | (36.6) |  | 5,251 |  | (36.9) |  | 6,821 |  | (38.4) |
|  | ≥35 | 3,846 |  | (24.0) |  | 3,457 |  | (26.9) |  | 4,075 |  | (28.7) |  | 5,430 |  | (30.6) |
|  | Body mass index, kg/m2 |  |  |  |  |  |  |  |  |  |  |  |  |  |  |  |
|  | <18.5 | 2,609 |  | (16.3) |  | 2,019 |  | (15.7) |  | 2,261 |  | (15.9) |  | 2,879 |  | (16.2) |
|  | 18.5 – <25 | 11,553 |  | (72.0) |  | 9,549 |  | (74.2) |  | 10,663 |  | (75.0) |  | 13,481 |  | (75.9) |
|  | ≥25 | 1,895 |  | (11.8) |  | 1,306 |  | (10.1) |  | 1,294 |  | (9.1) |  | 1,401 |  | (7.9) |
|  | Parity |  |  |  |  |  |  |  |  |  |  |  |  |  |  |  |
|  | Primipara | 6,377 |  | (39.7) |  | 4,932 |  | (38.3) |  | 6,408 |  | (45.1) |  | 9,171 |  | (51.6) |
|  | Multipara | 9,680 |  | (60.3) |  | 7,942 |  | (61.7) |  | 7,810 |  | (54.9) |  | 8,590 |  | (48.4) |
|  | Smoking status |  |  |  |  |  |  |  |  |  |  |  |  |  |  |  |
|  | Never | 8,545 |  | (53.2) |  | 7,715 |  | (59.9) |  | 8,977 |  | (63.1) |  | 11,765 |  | (66.2) |
|  | Former | 6,593 |  | (41.1) |  | 4,699 |  | (36.5) |  | 4,889 |  | (34.4) |  | 5,680 |  | (32.0) |
|  | Current | 919 |  | (5.7) |  | 460 |  | (3.6) |  | 352 |  | (2.5) |  | 316 |  | (1.8) |
|  | Passive smoking |  |  |  |  |  |  |  |  |  |  |  |  |  |  |  |
|  | No | 9,513 |  | (59.3) |  | 8,118 |  | (63.1) |  | 9,443 |  | (66.4) |  | 12,476 |  | (70.2) |
|  | Yes | 6,544 |  | (40.8) |  | 4,756 |  | (36.9) |  | 4,775 |  | (33.6) |  | 5,285 |  | (29.8) |
|  | Alcohol intake |  |  |  |  |  |  |  |  |  |  |  |  |  |  |  |
|  | Never | 5,497 |  | (34.2) |  | 4,459 |  | (34.6) |  | 4,735 |  | (33.3) |  | 5,813 |  | (32.7) |
|  | Former | 10,042 |  | (62.5) |  | 8,049 |  | (62.5) |  | 9,123 |  | (64.2) |  | 11,566 |  | (65.1) |
|  | Current | 518 |  | (3.2) |  | 366 |  | (2.8) |  | 360 |  | (2.5) |  | 382 |  | (2.2) |
|  | Physical activity |  |  |  |  |  |  |  |  |  |  |  |  |  |  |  |
|  | Q1 | 4,305 |  | (26.8) |  | 3,162 |  | (24.6) |  | 3,268 |  | (23.0) |  | 3,983 |  | (22.4) |
|  | Q2 | 3,826 |  | (23.8) |  | 3,303 |  | (25.7) |  | 3,675 |  | (25.9) |  | 4,520 |  | (25.5) |
|  | Q3 | 3,874 |  | (24.1) |  | 3,313 |  | (25.7) |  | 3,814 |  | (26.8) |  | 4,981 |  | (28.0) |
|  | Q4 | 4,052 |  | (25.2) |  | 3,096 |  | (24.1) |  | 3,461 |  | (24.3) |  | 4,277 |  | (24.1) |
|  | Quintile of folic acid intake, μg | | | |  |  |  |  |  |  |  |  |  |  |  |  |
|  | Q1 | 6,467 |  | (40.3) |  | 3,343 |  | (26.0) |  | 2,643 |  | (18.6) |  | 1,967 |  | (11.1) |
|  | Q2 | 4,188 |  | (26.1) |  | 3,629 |  | (28.2) |  | 3,799 |  | (26.7) |  | 3,743 |  | (21.1) |
|  | Q3 | 3,111 |  | (19.4) |  | 3,305 |  | (25.7) |  | 4,100 |  | (28.8) |  | 5,195 |  | (29.3) |
|  | Q4 | 2,291 |  | (14.3) |  | 2,597 |  | (20.2) |  | 3,676 |  | (25.9) |  | 6,856 |  | (38.6) |
|  | Energy intake |  |  |  |  |  |  |  |  |  |  |  |  |  |  |  |
|  | Q1 | 5,897 |  | (36.7) |  | 3,164 |  | (24.6) |  | 2,957 |  | (20.8) |  | 2,475 |  | (13.9) |
|  | Q2 | 4,362 |  | (27.2) |  | 3,626 |  | (28.2) |  | 3,791 |  | (26.7) |  | 4,002 |  | (22.5) |
|  | Q3 | 3,318 |  | (20.7) |  | 3,355 |  | (26.1) |  | 3,848 |  | (27.1) |  | 5,030 |  | (28.3) |
|  | Q4 | 2,480 |  | (15.4) |  | 2,729 |  | (21.2) |  | 3,622 |  | (25.5) |  | 6,254 |  | (35.2) |
|  | Marital status |  |  |  |  |  |  |  |  |  |  |  |  |  |  |  |
|  | Married | 15,364 |  | (95.7) |  | 12,446 |  | (96.7) |  | 13,764 |  | (96.8) |  | 17,326 |  | (97.6) |
|  | Single | 548 |  | (3.4) |  | 344 |  | (2.7) |  | 378 |  | (2.7) |  | 374 |  | (2.1) |
|  | Divorced or widowed | 145 |  | (0.9) |  | 84 |  | (0.7) |  | 76 |  | (0.5) |  | 61 |  | (0.3) |
|  | Highest education  level,y | |  |  |  |  |  |  |  |  |  |  |  |  |  |  |
|  | ≤12 | 6,784 |  | (42.3) |  | 4,380 |  | (34.0) |  | 4,141 |  | (29.1) |  | 4,437 |  | (25.0) |
|  | 12 – <16 | 6,376 |  | (39.7) |  | 5,604 |  | (43.5) |  | 6,332 |  | (44.5) |  | 8,082 |  | (45.5) |
|  | ≥16 | 2,897 |  | (18.0) |  | 2,890 |  | (22.5) |  | 3,745 |  | (26.3) |  | 5,242 |  | (29.5) |
|  | Highest education level of partner, y |  |  |  |  |  |  |  |  |  |  |  |  |  |  |  |
|  | ≤12 | 7,878 |  | (49.1) |  | 5,585 |  | (43.4) |  | 5,424 |  | (38.2) |  | 6,117 |  | (34.4) |
|  | 12 – <16 | 3,621 |  | (22.6) |  | 2,928 |  | (22.7) |  | 3,267 |  | (23.0) |  | 4,128 |  | (23.2) |
|  | ≥16 | 4,558 |  | (28.4) |  | 4,361 |  | (33.9) |  | 5,527 |  | (38.9) |  | 7,516 |  | (42.3) |
|  | Employed |  |  |  |  |  |  |  |  |  |  |  |  |  |  |  |
|  | No | 7,518 |  | (46.8) |  | 5,896 |  | (45.8) |  | 6,359 |  | (44.7) |  | 7,898 |  | (44.5) |
|  | Yes | 8,539 |  | (53.2) |  | 6,978 |  | (54.2) |  | 7,859 |  | (55.3) |  | 9,863 |  | (55.5) |
|  | Annual household income, million yen |  |  |  |  |  |  |  |  |  |  |  |  |  |  |  |
|  | <4 | 7,410 |  | (46.2) |  | 5,054 |  | (39.3) |  | 5,117 |  | (36.0) |  | 5,611 |  | (31.6) |
|  | 4 – <6 | 5,123 |  | (31.9) |  | 4,402 |  | (34.2) |  | 4,830 |  | (34.0) |  | 6,235 |  | (35.1) |
|  | ≥6 | 3,524 |  | (22.0) |  | 3,418 |  | (26.6) |  | 4,271 |  | (30.0) |  | 5,915 |  | (33.3) |
|  | Anti-bacteria medicine | |  |  |  |  |  |  |  |  |  |  |  |  |  |  |
|  | No | 14,323 |  | (89.2) |  | 11,441 |  | (88.9) |  | 12,677 |  | (89.2) |  | 15,922 |  | (89.7) |
|  | Yes | 1,734 |  | (10.8) |  | 1,433 |  | (11.1) |  | 1,541 |  | (10.8) |  | 1,839 |  | (10.4) |

|  | Supplemental Table3. Demographic and obstetric characteristics of participants (Natto:n=60,910) | | | | | | | | | | | | | | | |
| --- | --- | --- | --- | --- | --- | --- | --- | --- | --- | --- | --- | --- | --- | --- | --- | --- |
| Variable | | Fermented beans Intake (unit needed to be checked) | | | | | | | | | | | | |  |  |
|  |  | Q1 (0.0–1.7 g) | | |  | Q2 (3.3–5.4 g) | | |  | Q3 (10.7–12.5 g) | | |  | Q4 (16.1–600.0 g) | | |
|  |  | N (%) | | |  | N (%) | | |  | N (%) | | |  | N (%) | | |
| Subtotal | | 10,824 |  | (17.8) |  | 14,543 |  | (23.9) |  | 19,458 |  | (31.9) |  | 16,085 |  | (26.4) |
|  | Age, y |  |  |  |  |  |  |  |  |  |  |  |  |  |  |  |
|  | <25 | 1,148 |  | (10.6) |  | 1,441 |  | (9.9) |  | 1,356 |  | (7.0) |  | 874 |  | (5.4) |
|  | 25–<30 | 3,093 |  | (28.6) |  | 4,245 |  | (29.2) |  | 5,420 |  | (27.9) |  | 4,236 |  | (26.3) |
|  | 30–<35 | 3,673 |  | (33.9) |  | 5,125 |  | (35.2) |  | 7,337 |  | (37.7) |  | 6,154 |  | (38.3) |
|  | ≥35 | 2,910 |  | (26.9) |  | 3,732 |  | (25.7) |  | 5,345 |  | (27.5) |  | 4,821 |  | (30.0) |
|  | Body mass index, kg/m2 |  |  |  |  |  |  |  |  |  |  |  |  |  |  |  |
|  | <18.5 | 1,783 |  | (16.5) |  | 2,365 |  | (16.3) |  | 3,116 |  | (16.0) |  | 2,504 |  | (15.6) |
|  | 18.5 – <25 | 7,805 |  | (72.1) |  | 10,740 |  | (73.9) |  | 14,603 |  | (75.1) |  | 12,098 |  | (75.2) |
|  | ≥25 | 1,236 |  | (11.4) |  | 1,438 |  | (9.9) |  | 1,739 |  | (8.9) |  | 1,483 |  | (9.2) |
|  | Parity |  |  |  |  |  |  |  |  |  |  |  |  |  |  |  |
|  | Primipara | 5,178 |  | (47.8) |  | 6,708 |  | (46.1) |  | 8,208 |  | (42.2) |  | 6,794 |  | (42.2) |
|  | Multipara | 5,646 |  | (52.2) |  | 7,835 |  | (53.9) |  | 11,250 |  | (57.8) |  | 9,291 |  | (57.8) |
|  | Smoking status |  |  |  |  |  |  |  |  |  |  |  |  |  |  |  |
|  | Never | 6,649 |  | (61.4) |  | 8,685 |  | (59.7) |  | 11,936 |  | (61.3) |  | 9,732 |  | (60.5) |
|  | Former | 3,700 |  | (34.2) |  | 5,271 |  | (36.2) |  | 6,954 |  | (35.7) |  | 5,936 |  | (36.9) |
|  | Current | 475 |  | (4.4) |  | 587 |  | (4.0) |  | 568 |  | (2.9) |  | 417 |  | (2.6) |
|  | Passive smoking |  |  |  |  |  |  |  |  |  |  |  |  |  |  |  |
|  | No | 6,695 |  | (61.9) |  | 9,036 |  | (62.1) |  | 12,868 |  | (66.1) |  | 10,951 |  | (68.1) |
|  | Yes | 4,129 |  | (38.2) |  | 5,507 |  | (37.9) |  | 6,590 |  | (33.9) |  | 5,134 |  | (31.9) |
|  | Alcohol intake |  |  |  |  |  |  |  |  |  |  |  |  |  |  |  |
|  | Never | 4,068 |  | (37.6) |  | 5,034 |  | (34.6) |  | 6,293 |  | (32.3) |  | 5,109 |  | (31.8) |
|  | Former | 6,507 |  | (60.1) |  | 9,115 |  | (62.7) |  | 12,612 |  | (64.8) |  | 10,546 |  | (65.6) |
|  | Current | 249 |  | (2.3) |  | 394 |  | (2.7) |  | 553 |  | (2.8) |  | 430 |  | (2.7) |
|  | Physical activity |  |  |  |  |  |  |  |  |  |  |  |  |  |  |  |
|  | Q1 | 2,755 |  | (25.5) |  | 3,739 |  | (25.7) |  | 4,606 |  | (23.7) |  | 3,618 |  | (22.5) |
|  | Q2 | 2,602 |  | (24.0) |  | 3,678 |  | (25.3) |  | 5,084 |  | (26.1) |  | 3,960 |  | (24.6) |
|  | Q3 | 2,786 |  | (25.7) |  | 3,614 |  | (24.9) |  | 5,164 |  | (26.5) |  | 4,418 |  | (27.5) |
|  | Q4 | 2,681 |  | (24.8) |  | 3,512 |  | (24.2) |  | 4,604 |  | (23.7) |  | 4,089 |  | (25.4) |
|  | Quintile of folic acid intake, μg | | | |  |  |  |  |  |  |  |  |  |  |  |  |
|  | Q1 | 4,510 |  | (41.7) |  | 5,269 |  | (36.2) |  | 3,658 |  | (18.8) |  | 983 |  | (6.1) |
|  | Q2 | 2,679 |  | (24.8) |  | 4,168 |  | (28.7) |  | 5,702 |  | (29.3) |  | 2,810 |  | (17.5) |
|  | Q3 | 2,088 |  | (19.3) |  | 3,063 |  | (21.1) |  | 5,704 |  | (29.3) |  | 4,856 |  | (30.2) |
|  | Q4 | 1,547 |  | (14.3) |  | 2,043 |  | (14.1) |  | 4,394 |  | (22.6) |  | 7,436 |  | (46.2) |
|  | Energy intake |  |  |  |  |  |  |  |  |  |  |  |  |  |  |  |
|  | Q1 | 3,892 |  | (36.0) |  | 4,712 |  | (32.4) |  | 3,957 |  | (20.3) |  | 1,932 |  | (12.0) |
|  | Q2 | 2,764 |  | (25.5) |  | 4,199 |  | (28.9) |  | 5,465 |  | (28.1) |  | 3,353 |  | (20.9) |
|  | Q3 | 2,213 |  | (20.5) |  | 3,174 |  | (21.8) |  | 5,498 |  | (28.3) |  | 4,666 |  | (29.0) |
|  | Q4 | 1,955 |  | (18.1) |  | 2,458 |  | (16.9) |  | 4,538 |  | (23.3) |  | 6,134 |  | (38.1) |
|  | Marital status |  |  |  |  |  |  |  |  |  |  |  |  |  |  |  |
|  | Married | 10,344 |  | (95.6) |  | 13,994 |  | (96.2) |  | 18,874 |  | (97.0) |  | 15,688 |  | (97.5) |
|  | Single | 389 |  | (3.6) |  | 467 |  | (3.2) |  | 480 |  | (2.5) |  | 308 |  | (1.9) |
|  | Divorced or widowed | 91 |  | (0.8) |  | 82 |  | (0.6) |  | 104 |  | (0.5) |  | 89 |  | (0.6) |
|  | Highest education  level,y | | |  |  |  |  |  |  |  |  |  |  |  |  |  |
|  | ≤12 | 3,725 |  | (34.4) |  | 4,950 |  | (34.0) |  | 6,100 |  | (31.4) |  | 4,967 |  | (30.9) |
|  | 12 – <16 | 4,611 |  | (42.6) |  | 6,255 |  | (43.0) |  | 8,462 |  | (43.5) |  | 7,066 |  | (43.9) |
|  | ≥16 | 2,488 |  | (23.0) |  | 3,338 |  | (23.0) |  | 4,896 |  | (25.2) |  | 4,052 |  | (25.2) |
|  | Highest education level of |  |  |  |  |  |  |  |  |  |  |  |  |  |  |  |
|  | partner, y |  |  |  |  |  |  |  |  |  |  |  |  |  |  |  |
|  | ≤12 | 4,708 |  | (43.5) |  | 6,188 |  | (42.6) |  | 7,800 |  | (40.1) |  | 6,308 |  | (39.2) |
|  | 12 – <16 | 2,443 |  | (22.6) |  | 3,454 |  | (23.8) |  | 4,374 |  | (22.5) |  | 3,673 |  | (22.8) |
|  | ≥16 | 3,673 |  | (33.9) |  | 4,901 |  | (33.7) |  | 7,284 |  | (37.4) |  | 6,104 |  | (38.0) |
|  | Employed |  |  |  |  |  |  |  |  |  |  |  |  |  |  |  |
|  | No | 4,865 |  | (45.0) |  | 6,290 |  | (43.3) |  | 8,930 |  | (45.9) |  | 7,586 |  | (47.2) |
|  | Yes | 5,959 |  | (55.1) |  | 8,253 |  | (56.8) |  | 10,528 |  | (54.1) |  | 8,499 |  | (52.8) |
|  | Annual household income, |  |  |  |  |  |  |  |  |  |  |  |  |  |  |  |
|  | million yen |  |  |  |  |  |  |  |  |  |  |  |  |  |  |  |
|  | <4 | 4,438 |  | (41.0) |  | 5,751 |  | (39.5) |  | 7,101 |  | (36.5) |  | 5,902 |  | (36.7) |
|  | 4 – <6 | 3,531 |  | (32.6) |  | 4,870 |  | (33.5) |  | 6,701 |  | (34.4) |  | 5,488 |  | (34.1) |
|  | ≥6 | 2,855 |  | (26.4) |  | 3,922 |  | (27.0) |  | 5,656 |  | (29.1) |  | 4,695 |  | (29.2) |
|  | Anti-bacteria medicine | |  |  |  |  |  |  |  |  |  |  |  |  |  |  |
|  | No | 9,794 |  | (90.5) |  | 12,979 |  | (89.3) |  | 17,336 |  | (89.1) |  | 14,254 |  | (88.6) |
|  | Yes | 1,030 |  | (9.5) |  | 1,564 |  | (10.8) |  | 2,122 |  | (10.9) |  | 1,831 |  | (11.4) |
